# Delving deeper into technological innovations to understand differences in rice quality

**DOI:** 10.1186/s12284-015-0043-8

**Published:** 2015-01-29

**Authors:** Mariafe Calingacion, Lu Fang, Lenie Quiatchon-Baeza, Roland Mumm, Arthur Riedel, Robert D Hall, Melissa Fitzgerald

**Affiliations:** Grain Quality and Nutrition Centre, International Rice Research Institute, DAPO 7777, Metro Manila, Philippines; Laboratory of Plant Physiology, Wageningen University and Research Centre, Wageningen, The Netherlands; School of Agriculture and Food Science, University of Queensland, St Lucia, 4072 Queensland, Australia; Department of Crop Sciences, College of ACES, University of Illinois at Urbana-Champaign, Urbana, IL USA; BU Bioscience, Plant Research International, Wageningen University and Research Centre, Wageningen, The Netherlands

**Keywords:** Metabolomics, Rice, Climate change, Consumer acceptance, Eating quality

## Abstract

Increasing demand for better quality rice varieties, which are also more suited to growth under sub-optimal cultivation conditions, is driving innovation in rice research. Here we have used a multi-disciplinary approach, involving SNP-based genotyping together with phenotyping based on yield analysis, metabolomic analysis of grain volatiles, and sensory panel analysis to determine differences between two contrasting rice varieties, Apo and IR64. Plants were grown under standard and drought-induced conditions. Results revealed important differences between the volatile profiles of the two rice varieties and we relate these differences to those perceived by the sensory panel. Apo, which is the more drought tolerant variety, was less affected by the drought condition concerning both sensory profile and yield; IR64, which has higher quality but is drought sensitive, showed greater differences in these characteristics in response to the two growth conditions. Metabolomics analyses using GCxGC-MS, followed by multivariate statistical analyses of the data, revealed a number of discriminatory compounds between the varieties, but also effects of the difference in cultivation conditions. Results indicate the complexity of rice volatile profile, even of non-aromatic varieties, and how metabolomics can be used to help link changes in aroma profile with the sensory phenotype. Our outcomes also suggest valuable multi-disciplinary approaches which can be used to help define the aroma profile in rice, and its underlying genetic background, in order to support breeders in the generation of improved rice varieties combining high yield with high quality, and tolerance of both these traits to climate change.

**Electronic supplementary material:**

The online version of this article (doi:10.1186/s12284-015-0043-8) contains supplementary material, which is available to authorized users.

## Background

Asia currently faces two major challenges that are predicted to have significant impacts on food security in the region: rapid and significant population growth (United Nations 2014), and climate change (IPCC 2014). The Asian population is growing at a much faster rate than those in other regions, and is predicted to peak by 2050 (UnitedNations 2014). Rice is the staple food of Asia, and to protect Asia against food shortages in the future, it is imperative that rice improvement programs develop new varieties with much higher yield. However, finding an acceptable solution is more complex than increasing yield potential alone.

Socioeconomic patterns within the Asian population are changing rapidly. While research is helping to meet the United Nations Millennium Development goals, by lifting people out of poverty (Fanzo and Pronyk 2011), Asia is currently undergoing economic transformation (Kharas and Gertz 2010), and is predicted to become home to the largest middle class by 2030. Following from this economic growth, it is expected that the majority of Asians will have more discretionary income and greater financial capacity to make food choices based on assessment of quality and safety (Goodman and Robison 2013). Therefore, the impact of population growth in Asia, on both rice production and rice improvement programs, is that new high-yielding rice varieties must contain the quality traits that increasingly-discriminatory consumers in different Asian markets require (Calingacion et al. 2014). Combining yield and quality in rice is not trivial, because the tools for measuring quality are unable to discriminate in a manner that explains the sensory experiences of aroma, taste, flavour and texture (Daygon and Fitzgerald 2013; Fitzgerald et al. 2009).

Further challenging the need for more and better rice, the effects of climate change are predicted to impact most strongly in the latitudes and longitudes of relevant Asia (IPCC 2014). Climate change is predicted to lead to more drought events, higher temperatures (Peng et al. 2004), and more unpredictable weather patterns (Knutson and Tuleya 2004; Trenberth 2005). In anticipation of this dramatically changing environment, rice improvement programs now include breeding and selection for climate-ready varieties of rice (Zeigler and Barclay 2008). The breeding and domestication of rice has created variation among genotypes that enables cultivation in a number of less favourable environments, including salinity-affected coastal areas, water-scarce upland areas, and lowland monsoonal regions that are prone to inundation and flood (Mackill et al. 2012). These adaptations demonstrate that the species houses genes that enable a high degree of plasticity, and thus permit productive growth in different environments (Wassmann et al. 2009). Identifying the genetic basis of different stress tolerances, in order to introgress causal genes into elite varieties is a research priority. Recent examples of gains in managing the need for stress-tolerant rices are uncovering (i) genes for submergence tolerance for rice grown in monsoonal regions (Xu et al. 2006), and (ii) genes for drought tolerance for varieties grown in conditions of water scarcity or drought (Kumar et al. 2014).

New rice varieties with tolerance to abiotic stresses offer a clear yield advantage to farmers. However, this does not always translate into profit because, next to agronomic traits, a major driver of widespread uptake of a new variety is not farmer adoption, but consumer acceptance (Calingacion et al. 2014; Daygon and Fitzgerald 2013; Boualaphanh et al. 2011; Champagne et al. 2010; Fitzgerald et al. 2009). A clear example of this exists in the case of the two varieties Apo (also known as PSBRc 9) and IR64. Apo repeatedly gives good yield under drought conditions (Venuprasad et al. 2007); it has been released in several drought-prone areas of Asia, but has not been widely adopted, because consumers consider it to have poor eating quality (Fitzgerald et al. 2009; Champagne et al. 2010). IR64, on the other hand, is susceptible to many abiotic stresses, including drought (Venuprasad et al. 2007), but regardless, it is widely accepted and has been grown annually on over a million hectares in major rice-producing countries since its release in 1985 (Fitzgerald et al. 2009).

Apo and IR64 have been assessed in a paired comparison by a sensory evaluation panel, comprising another 11 similarly matched pairs of rice varieties, where both varieties in each pair had similar physico-chemical traits of quality, but differed in their consumer acceptance/popularity (Champagne et al. 2010). The flavour of Apo rice was found to be dominated by sewer/animal, astringent, and water-like metallic notes, whereas IR64 was predominantly characterised by sweet and corn notes (Champagne et al. 2010). The compounds that individually, or in combination, are responsible for such flavours and aromas have not yet been identified. In order to assist rice improvement programs to develop varieties that meet consumer taste and flavour requirements as well as meeting the need for increased yield, newly developed phenotyping techniques such as metabolomics can be used to identify these compounds of taste and flavour in foods (Calingacion et al. 2012; Hall et al. 2008).

Our objectives here were to (i) confirm that flavour notes previously reported by Champagne et al. (2010) for Apo and IR64 grains are reproducible, and are therefore robust characteristics of the two varieties; (ii) determine how water scarcity affects these flavour notes, because the cultivation of Apo germplasm was originally developed for water-scarce environments; and (iii) identify a panel of target compounds with low odour threshold that differ between Apo and IR64, and investigate their association with the phenotypic aromas previously reported. To assemble the most detailed picture of these two important rice varieties, and to see how key traits are influenced by water availability, we have used a highly multi-disciplinary approach involving phenotyping (metabolomics, taste panel sensory analysis, yield measurements) supported by advanced multivariate statistical analyses.

## Results

### Response to drought treatment

**Figure 1:**
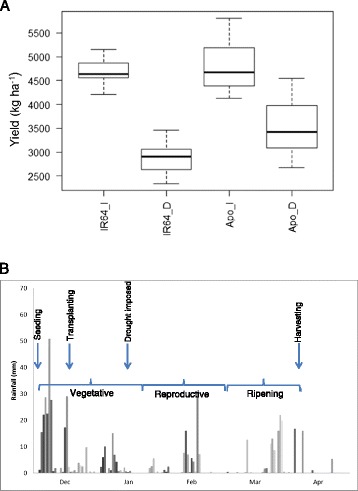
**A.** **Yields (kg ha** ^**−1**^ **) of Apo and IR64 rices grown under irrigated and drought conditions (Dry Season 2012), N=48.** **B**. Rainfall (mm) at the Experimental Station of International Rice Research Institute, Philippines during the growth of the rice samples (Dry Season 2012).

Drought significantly lowered yield for Apo and IR64 by 27% and 38%, respectively (effect of removing treatment from the model: Χ^2^_1_ = 20.855, p = <0.0001) (Figure 1A). There was a significant difference in overall yield between the two varieties (effect of removing variety from the model: Χ^2^_1_ = 6.5484, p = <0.0105). The mean yield of IR64 was lower than that of Apo, but it showed no statistically significant interaction between genotype and treatment, meaning that neither variety was significantly more affected by treatment than the other. Over the duration of the experiment, there was 3.5 mm average rainfall per day, with some rain falling during grain-filling (Figure 1B), but the artificial drought imposed on the plots by draining at panicle initiation and just before flowering, was enough to significantly affect yield (Figure 1A).

### Sensory evaluation

The sensory panel reproducibly detected aromas of sweet taste, corn and sweet aromatic flavours in the grains of IR64 from irrigated plots, and water-like metallic, astringent, sour silage, sewer animal and hay-like/musty aromas in Apo grains from irrigated plots (Table 1). When comparing the effects of drought within varieties, no difference in aroma was detected for grains of Apo (Table 1). For the two IR64 samples, the levels of corn and sweet aromatic aromas were similar in both treatments. However, higher levels of sweet taste and hay-like/musty were perceived in IR64 from the irrigated than the drought treatment. In addition, water-like metallic, astringent and sour/silage aromas were detected in IR64 grains from the drought treatment.

**Table 1:** **Comparison of flavour attributes in Apo and IR64 from irrigated and drought treatments with sensory qualities/attributes as previously reported by Champagne et al. (** 2010 **)**

**Flavour**	**Apo** ^**a**^	**IR64** ^**a**^	**Apo**		**IR64**	
			**Irrigated**	**Drought**	**Irrigated**	**Drought**
Sweet taste	+	++	+	+	++	+
Corn		+			+	+
Sweet aromatic		+			+	+
Astringent	++	+	+	+		+
Water like metallic	++	+	++	++		+
Sewer/animal	++		++	++		
Sour/silage	++		+	+		+
Hay-like/musty			++	++	++	+

^a^Adapted from Champagne et al. (2010).

Eight panelists indicated the strength of the perceived aromatic characteristic of the sample using a 3 point scale.

### Analysis of headspace samples by GCxGC-TOFMS

**Figure 2:**
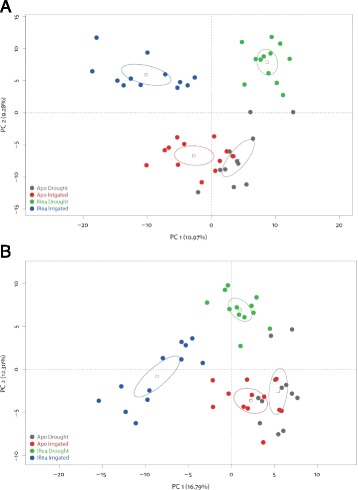
**A.** **PCA of metabolites detected in 12 biological samples of each Apo and IR64 from irrigated and drought treatments, N=48.** **B**. PCA of metabolites with known flavour and low odour threshold detected in Apo and IR64 grown in irrigated and drought treatments, N=48.

We detected 187 volatile compounds in Apo and IR64 (Figure 2A). Principal components analysis (PCA) extracted the main axes of variation in the concentration of volatile compounds detected in Apo and IR64 from irrigated and drought treatments. PC1 explained 11% of the variation in metabolite concentrations, with IR64 forming two distinct clusters corresponding to water availability during growth (Figure 2A). Apo from both irrigation treatments clustered closely and showed considerably less variation in PC1 than IR64. Our linear mixed model analysis found no significant interactions between treatment and block effects (effect of removing block × variety interaction from the model: Χ^2^_3_ = 5.1799, p = 0.1591) in PC1, so this term was removed from the model before assessing the fixed treatment effects, but we retained the random block component to partition out variances derived from the experimental design. Critically, despite discerning no differing effects on yields between the two varieties, we found a substantive part of the variation in metabolites on PC1 was associated with variation in the differing treatment responses of the two rice varieties (effect of removing two-way interaction, variety × treatment, from the model: Χ^2^_1_ = 22.271, p = <0.0001).

PC2, encapsulating 9% of the total variation modelled, further distinguished samples according to their variety: all Apo samples loaded negatively on PC2, all IR64 samples loaded positively. Our linear mixed model found no variation between varieties among blocks (effect of removing block × variety interaction from the model: Χ^2^_3_ = 0, p = 1). Variation in scores for PC2 was found to be associated solely with differences between the two rice varieties (effect of removing variety from the model: Χ^2^_1_ = 90.93, p = <2.2e-16).

A second PCA (Figure 2B) of selected compounds with aromatic descriptors and low odour threshold compounds, showed some characteristics in common with Figure 2A. PC1, explaining 17% of the total variation in low odour threshold metabolites, described broad separation of IR64 grown in irrigated conditions, with negative scores in this dimension for all samples. IR64 grown under drought conditions showed both positive and negative scores on PC1, where both Apo treatments were closely clustered with predominantly positive scores. PC2 (12.5%) allocated positive scores to the IR64 drought-treated samples, negative scores to both Apo sample groups, and widely scattered scores for IR64 grown in irrigated conditions whose mean score values were nominally negative on PC2 (Figure 2B).

Sparse Partial Least Squares-Discriminant Analysis (sPLS-DA) identified 20 compounds for each direction through the data that discriminated between treatment groups (Table 2). The first axis discriminated samples from the IR64 irrigated treatment, the second discriminated IR64 drought samples from the two Apo treatment classes, while the third described those compounds that differed between the two treatments applied to Apo.

**Table 2:** **Discriminating compounds, of known flavour and low odour threshold, in Apo and IR64 grown in irrigated and drought treatments**

**First axis**		**Second axis**		**Third axis**	
**Compound**	**Loading**	**Compound**	**Loading**	**Compound**	**Loading**
Limonene	−0.391	Methylbenzoate	−0.406	1,2,3,4-Tetramethyl benzene	0.434
n-Propylbenzene	−0.372	Camphene	−0.401	Tridecane	0.425
1-Ethyl-3-methylbenzene	−0.358	Benzonitrile	−0.379	Phenethylacetate	0.366
Indole	0.338	Tetradecane	0.368	Benzophenone	−0.361
2-Heptanone	−0.297	Phenol	−0.334	3-Methylbenzaldehyde	0.262
2-Heptenal	−0.249	Isopropyldodecanoate	−0.227	Pentadecanoic acid	0.243
Hexanal	−0.233	Decanal	−0.200	Isomethyl ionone	0.225
1-Hepten-3-ol	−0.231	Benzaldehyde	−0.199	Caryophyllene	−0.200
2-Pentylfuran	−0.216	Octanal	−0.194	Naphthalene	0.199
Methylstyrene	−0.201	Heptanal	−0.193	o-Cymene	0.194
Ethyl octanoate	−0.175	1-Propene-1-thiol	0.167	Benzaldehyde	−0.161
2-Propylfuran	−0.173	Acetophenone	−0.102	1-Hexanol	0.139
α-Phellandrene	−0.168	4-Hydroxy-4-methyl-2-pentanone	−0.096	α-Farnesene	0.122
o-Cymene	−0.118	2-Pentylfuran	0.093	2-Methylheptane	0.073
β-Ocimene	−0.107	2-Methylheptane	0.091	2-Ethylhexanal	−0.045
2-Octenal	−0.071	Carvone	−0.087	p-Xylene	−0.041
2-Butylfuran	−0.027	Hexanal	0.065	4-Hydroxy-4-methyl-2-pentanone	0.036
1,3-Dimethylbenzene	−0.014	2,4-Hexadien-1-ol	−0.042	Undecane	0.028
Undecane	−0.009	Pentadecanoic acid	−0.009	Phenol	0.019
α-Pinene	−0.001	Indole	0.007	α-Phellandrene	0.006

These compounds have been extracted from each of the three axes of a sparse Partial Least Squares-Discriminant Analysis.

## Discussion

As climate change exerts its influence on rice-producing countries, it is essential that science delivers new phenotyping tools to support the development and release of new varieties of rice for new and challenging environments. These new varieties must combine stress tolerance for both yield and grain quality in order to ensure consumer acceptability (Calingacion et al. 2014). The difficulty in combining both traits and then adding stress tolerance is demonstrated by the variety Apo, which has been released for drought tolerance (Espino and Cinense 2010) but its cooking and eating qualities are unacceptable, in spite having identical physico-chemical properties to IR64 and other popular varieties.

Current tools used to evaluate rice quality are not sufficiently discriminating (Calingacion et al. 2014; Daygon and Fitzgerald 2013; Fitzgerald et al. 2009). Therefore breeding programs have used a strategy to create stress-tolerant versions of already popular varieties of rice using genetic pyramiding techniques (Mackill et al. 2012). These ‘upgraded’ varieties, for example the *sub*1 series (Mackill et al. 2012) can be grown successfully in more challenging environments. However there is still a need to understand and control cooking and eating quality to assure these upgraded varieties continue to be accepted by consumers (Sundaram et al. 2008; Win et al. 2012; Septiningsih et al. 2009), and to make further gains in breeding targets. As genotyping technologies develop, tools enabling genetic selection become more accessible, and simultaneously, the need to push through current yield barriers becomes more pressing. Therefore bringing new science to rice quality to identify traits that consumers value is a more purposeful pathway forward in the longer term, than simply upgrading current varieties with stress tolerant genes.

In the present study, two rice varieties, the unpopular, drought-tolerant Apo and the highly popular, drought-susceptible IR64, were evaluated for yield and drought tolerance, sensory flavour and volatile metabolites in the grain, and finally genetic differences. Data obtained were used to (i) identify a panel of compounds that may be linked to the cooking and eating quality of the rices, (ii) determine the effect of drought stress on the compounds; and (iii) evaluate the potential of using a mapping population developed from these two varieties to enable future identification of genes that underlie the aromas emitted during cooking and consumption of the rices. The ultimate goal envisaged is that this work will lead to the identification of quantitative trait loci (QTLs) for key quality-related traits which will then provide new tools for breeding new, improved varieties with enhanced sensory and agronomic characteristics.

### Response to drought treatment

Drought during the reproductive stage can reduce spikelet fertility, panicle exsertion and source-sink interactions which all decrease yield (Wassmann et al. 2009). Yield can therefore be used as a proxy to measure the degree of drought tolerance (Kumar et al. 2008). Several studies have demonstrated the drought tolerance of Apo (Venuprasad et al. 2007; Venuprasad et al. 2009; Venuprasad et al. 2012 and susceptibility of IR64 {Venuprasad et al. 2007 #21; Kumar et al. 2008; Guan et al. 2010; Vikram et al. 2011; Ghimire et al. 2012; Palanog et al. 2014). However, in our experiment, the yield of both varieties under drought was reduced, and surprisingly there was no statistical difference between the two varieties (Figure 1A, Additional file 1: Table S1). Furthermore, drought significantly affected the aromas and flavours of the grain of IR64, but it did not have such an effect on the grain of Apo (Figures 2A and B).

### Sensory analysis of the grains

Consumer acceptance of a variety of rice is driven initially by the physical appearance of the grains (Fitzgerald et al. 2009), and further cemented by, first, the cooking and second, the eating quality of rice (Cramer et al. 1993). Characteristics of cooking rice that are important to consumers are (i) the aromas generated by the processes of cooking rice, for example the sweet and floral aromas emitted when jasmine rice is cooking; (ii) cooking time; and (iii) the appearance of the cooked rice when ready for serving and eating (Bett-Garber et al. 2012). Eating quality describes the aroma, flavour, taste and texture of the rice in the mouth (Fitzgerald et al. 2009). Differences in the aroma of Apo and IR64 during and after cooking are likely to explain much of the differences in consumer acceptance of the two varieties. The aroma of IR64 grains has previously been evaluated as being high in sweet taste, and low in astringent and water-like metallic attributes, whereas Apo was described as high in sewer/animal, water-like metallic, astringent and sour/silage and low in sweet taste (Champagne et al. 2010). In that study, the rice had been grown in irrigated conditions in the same country as the rice evaluated in the present research, but 4 years earlier than this study, and in a different province. The results presented here for the samples from irrigated treatments accord well with those of the previous study. In addition, we found high levels of hay-like/musty flavours in Apo that were not previously reported by Champagne et al. (2010). Slight differences between our results with those of previous reports may derive from the harvest and storage conditions of the samples tested, but in general, the aromatic qualities of each variety are reproducible.

Sensory analysis and the volatile profile of IR64 were both affected by the drought treatment. Biochemical changes in the grain may explain much of this, either due to changes in gene expression in the grain, or changes to the regulation of source-sink transport from the plant to the grain. In IR64 grains from the drought treatment, sweetness was decreased and a water-like metallic flavour was detected which was absent in grains from the irrigated treatment. Though we cannot demonstrate the role of the underlying metabolic processes in this study, we have linked the stress caused by drought to changes in the metabolite pattern, and therefore the flavour profile of IR64. Samples of Apo from both irrigated and drought fields were indistinguishable in their characterisation by water-like metallic, sewer/animal, and hay-like/musty aromas at high levels. One previous study has investigated the effect of drainage on the sensory aroma of rice and the variety used in that study showed no difference in flavour/aroma compounds (Champagne et al. 2005). Our data suggest that a response to moisture stress in the metabolomic profile of the grain is variety dependent, as is often observed in crops e.g., fruit response to water stress that is cultivar dependent (Giné Bordonaba and Terry 2010). Understanding different varietal responses and determining the basis of stress response is important in developing climate-ready varieties.

### Sensory analysis correlates with volatile metabolite profiles

Our analysis has highlighted variety x treatment interactions, with IR64 grains showing greater environmental sensitivity than Apo grains. The differences in IR64, due to differences in water availability, highlight how water stress not only affects grain yield, but also the metabolites present in the grains, and we found this to be reflected in the responses of the sensory panel. By contrast, volatile metabolites present in Apo showed less variation between the water treatments, an outcome also reflected in the uniform characterisation of Apo rice by the sensory panel. The clear differences between Apo and IR64 in their volatile profiles, which are supported by the findings of the sensory panel, reinforces that metabolite profiling is indeed capable of distinguishing between varieties (Calingacion et al. 2012), providing a tool to identify and potentially quantify important compounds of rice quality.

Volatile compounds can only be considered important in rice quality if the odour threshold, which defines the lowest concentration of a given compound that humans can perceive, is low (Leonardos et al. 1969). Of the 187 compounds detected, 65 have been putatively identified as aromatic with a low odour threshold. The PCA of this shortlist of 65 closely reflected the PCA of all 187 compounds, suggesting that much of the variation between varieties was captured with less, but more relevant data. Statistical analysis was further able to identify those flavour compounds that most discriminate between Apo and IR64. Samples of IR64 from the irrigated treatment formed a distinct cluster in the first axis, with indole as the compound most strongly correlated with that dimension (Table 2). Indole has a sweet flavour and is used at low concentrations to form synthetic sweet floral aromas (Jezussek et al. 2002), so it is a promising candidate for the sweeter taste perceived in the grains of IR64 from the irrigated treatment, and in agreement with previous reports for rice grown in France (Maraval et al. 2008).

Samples of IR64 from the drought treatment were discriminated by strong loadings of octanal and decanal, which are both described as having a low odour threshold of fruity or waxy odours (Jezussek et al. 2002; Yang et al. 2008). Hexanal, heptanal, octanal and decanal were also present, as were their primary alcohols and several alkanes, possibly representing oxidation ladders (Monsoor et al. 2004), with beneficial outcomes for the aromatic profile of the rice.

Sensory differences between irrigated and drought samples of IR64 identified an astringent aroma in the drought samples (Table 1). Phenol is present in IR64 drought and Apo grains (Table 2), and this is a compound commonly associated with astringency (Bahar and Altug 2009). Undesirable aromas such as sewer animal and sour/silage were found in Apo alone, often/typically associated with the presence of propene thiol and ocimene (O’Neill and Phillips 1992; Loper et al. 1971), both of which were detected in Apo. Hay-like/musty off-flavour aromas were most strongly detected by the sensory evaluation in drought-affected IR64 and both samples of Apo. Hexanal and heptanal could explain those aromas (Lam and Proctor 2003). The strong similarity between the sensory data of the samples and the pattern of loadings in Figure 2B indicates that delving deeper into metabolomics to explain aroma could lead to the development of a new suite of tools to select for aromatic profiles.

Our study again shows that over a hundred volatile metabolites are present in rice (Laguerre et al. 2007; Bryant and McClung 2011; Mathure et al. 2014), and while only a handful may be key to characterizing and defining rice aroma (Buttery et al. 1988) (Buttery et al. 1988; Jezussek et al. 2002; Yang et al. 2010; Calingacion et al. 2012), their relative importance needs to be better understood to enable breeders to select for this trait. Little is known about the genetic basis of aroma in rice, or the inheritance patterns of key flavour compounds, aside from 2-acetyl-1-pyrroline, which is associated with the popcorn-like aroma of jasmine and basmati rices (Bradbury et al. 2005; Kovach et al. 2009; Chen et al. 2008). Here we have taken substantial steps towards identifying additional specific compounds associated with favourable and unfavourable consumer responses to rice aromatic quality. With a better understanding of the flavour traits that influence rice acceptance, and identifying specific compounds that drive this acceptance, we gain the potential to take advantage of the genetic difference between Apo and IR64 (Additional file 2: Figures S1) for discovery of causative loci. Development of a mapping population between Apo and IR64 could facilitate the identification of QTLs associated with specific components of rice aroma. This work makes it possible to develop varieties that take the favourable aromatic/flavour characteristics of one variety and at the same time select for the drought tolerant traits of another.

## Conclusions

A panel of compounds identified here that are strongly associated with the pleasant cooking and eating experience of IR64 are indole, octanal, and decanal which give sweet, fruity aromas, while Apo is identified with off-flavour compounds such as propene thiol, ocimene, heptanal and hexanal which give sewer/animal, sour/silage and hay-like/musty aromas respectively. These compounds all have low odour thresholds and associate with the aromas detected by sensory analysis in this and previous studies. Identification of the genes underlying these flavour notes, requires identifying QTLs associated with the specific compounds that appear to drive consumer responses. Such QTLs will greatly improve the efficiency and precision of market-driven breeding programs.

## Methods

### Plant material

Apo and IR64 were planted at the Experimental Station of the International Rice Research Institute, Philippines in the dry season of 2012. For each variety, 100 plants, at 1 plant per hill, were planted at 15 cm within and between rows in 3 blocks under irrigated and 3 blocks under drought conditions, with 4 replicated plots in each block. Inorganic fertiliser nitrogen: phosphorus: potassium (NPK) was applied to the field before transplanting at a ratio of 40:40:40 kg ha^−1^ (NPK), and the plants were top-dressed with urea 30 and 55 days after transplanting at a level of 30:0:0 kg ha^−1^. Drought stress was artificially imposed by draining the field when plants had been assessed to have achieved maximal tillering, so that water stress coincided with the reproductive stage of the plant. Irrigated plots were maintained at a water level of approximately 5 cm until harvest, at which time they were drained. Mature grains from all 100 plants were harvested to determine total yield. The grains were dried in an oven until a moisture content of 12-14% was reached and stored at 21°C storage room until analysis. Grains were dehulled (Otake FCY2 Dehusker, Oharu, Japan), polished (test tube mill) and cryo-ground (2010SPEX SamplePrep Geno/Grinder) for 3 min at 1750 rpm. We compared yields of the four treatment classes (2 varieties × 2 treatments) in a two factor linear mixed effects model that assessed the presence of variety x treatment interactions.

### Sensory evaluation of flour

Eight trained panelists participated in this study. Rice samples were prepared by placing rice flour (1 g) in screw capped vials and heating in a water bath at 80°C for 10 min. Rice was presented in this way to replicate the way the sample and its volatile compounds were later presented for headspace analysis by gas chromatography. Samples were presented to panelists unidentified and in randomised order. Panelists opened the lid of the vial carefully and smelt the aroma of the heated rices. Panelists then scored the intensity or absence of eight flavour notes on a 3-point intensity scale. The flavour notes considered were: sweet taste, corn, sweet aromatic, astringent, water-like metallic, sewer/animal, sour/silage and hay-like/musty (Champagne et al. 2010). Panelists were free to record additional aromas they detected.

### Headspace analysis using two-dimensional gas chromatography time of flight-mass spectrometry (GCxGC TOF-MS)

#### Headspace extraction

Rice flour (1 g) was placed in a sealed vial and allowed to equilibrate overnight at room temperature. The sample was then heated at 80°C for 10 min while being agitated at 500 rpm in an agitator of autosampler of GCxGC TOF-MS. Volatile compounds in the headspace (1 mL) were collected in a syringe and 1.5 mL was injected in a GCxGC TOF-MS system consisting of an Agilent 7890 gas chromatograph (Agilent Technologies, Palo Alto, CA) and Pegasus IV TOF-MS mass spectrometer (LECO, St Joseph, MI). The primary column used was RXI-5sil (29 m × 0.25 mm id × 0.25 μm film thickness) while the secondary column was RxI-17 (1.7 m × 0.1 mm id × 0.18 μm film thickness). The secondary column was placed inside the secondary oven after the thermal modulator. The flow rate of the helium carrier gas was set to a constant flow of 1 mL min^-1^. The primary column was set at an initial temperature of 45°C for 1 min, then ramped at 10°C min^−1^ to 250°C. The secondary column was programmed with an initial temperature of 60°C for 1 min which was then increased to 250°C at a rate of 10°C min^−1^. The thermal modulator was set at 70°C then ramped to 260°C at a rate of 10°C min^−1^ with a 5 s modulation period. The MS mass range was 35–400 m/z with an acquisition rate of 200 spectra s^−1^. The ion source temperature was set at 250°C and the detector voltage was 1.5 kV with electron energy of -70 eV and the temperature of the transfer line was 280°C. Headspace of a standard mixture of known compounds (benzene, toluene, ethylbenzene, p-xylene) was also injected as a quality control sample to check if peak identification is accurate and reproducible.

#### Data processing

The raw GCxGC data were processed using ChromaTOF software (version 4.5, LECO, St Joseph, MI). We used the automated peak finding and spectral deconvolution algorithm with baseline offset of 0.5 and signal to noise ratio of 6. Automated retention time alignment was also performed using the statistical comparison function in ChromaTOF. All samples were divided among the two water treatment classes, then data were aligned within and between the classes. Only the compounds that were present in more than 50% of the samples in a class were considered. Mass spectra were compared and matched against the NIST 2011 mass spectral library (http://www.NIST.gov).

#### Statistical analysis

All statistical analyses were done in R (RCoreTeam 2014), using a number of packages. First, we assessed treatment effects and interactions on yield, using a simple univariate model. For our multivariate data, to remedy the skew of several variables, they were first log transformed. To moderate the influence of large-scale variation among some compounds, and thereby allow smaller-scale variation in other metabolites to play a greater role in the overall characterisation of rice aromatic qualities, we Pareto scaled all data (van den Berg et al. 2006): for each variable, data was centred on a mean of zero and the variance scaled by dividing by the square root of the standard deviation for each variable (R package: ‘MetabolAnalyze’, (Gift et al. 2010)). We used Principal Components Analysis (PCA) (R package: ‘FactoMineR’, (Husson et al. 2013)) to extract the first two dimensions in our data that explained the greatest amount of variation. To support our description of the treatment classes by PCA, we extracted the PC scores for the 48 individual samples on these first two dimensions, and treated these condensed descriptions of the principal variation in the data as response variables in a linear model. Since our data included experimental blocks nested within growing condition (irrigated vs. drought), in all analyses we used linear mixed effects modelling to partition out the random effects from analysis of treatment effects on the metabolic profile encapsulated in the first two principal components (R package: ‘lme4’, (Bates et al. 2014)); as recommended for mixed models, we used Kenward-Rogers estimation of the denominator degrees of freedom (Saxton 2004). We established the significance of effects in models in the usual manner (Quinn and Keough 2002): log-likelihood ratio tests compared a full model of treatment factors, and the random experimental effects, with a nested model omitting successive effects.

In the second part of the analysis, we looked more closely at a shortlist of compounds of known low odour threshold, as these have greatest influence on rice quality and consumer preference. We created a data set comprising 65 compounds of the original 187, and again explored the treatment effects on chemical composition using PCA of those compounds that are known to influence aroma.

High dimensional data, such as is generated by GCxGC MS, is typically explored using PCA; PC loadings are one method to extract which compounds show the greatest variation, and therefore may be having greatest influence in distinguishing groups. However, high dimensional data presents a challenge in discerning which variables are more important, and the criteria for acceptance or rejection of putatively important contributions can be arbitrary and less reliable when based on the, typically small, sample sizes of these methods. Here, using a method that gives greater statistical underpinning to the choice of important variables, we used sPLS-DA (R package ‘mixOmics’; (Dejean et al. 2013)), to identify those compounds likely to be most influential in discriminating between treatment effects on the two rice varieties in k-1 dimensions, i.e., three in this instance. We constrained the model to identify the 20 compounds, each with a low odour threshold, that were most influential in discriminating between the treatment groups, for each dimension in turn, extending to three dimensions, which were then extracted from the sPLS-DA model.

## Additional files

Additional file 1: Table S1. Log-likelihood ratio tests: effect of removing fixed effect terms for linear mixed models of yield (A), PC1 (B) and PC2 (C). Additional file 2: Figure S1. Pedigree tree of (A) Apo (IRRI 132) and (B) IR64 showing no common parents in either background for three generations immediately prior to the final cross (http://www.irri.org/tools-and-databases/international-rice-information-system).
