# Supplementary material for: Delving deeper into technological innovations to understand differences in rice quality
Source: Rice (N Y). 2015 Jan 29;8:6. doi: 10.1186/s12284-015-0043-8 (PMC4883128; doi:10.1186/s12284-015-0043-8)
Supplement: Additional file 1: Table S1. — Log-likelihood ratio tests: effect of removing fixed effect terms for linear mixed models of yield (A), PC1 (B) and PC2 (C). [file 12284_2015_43_MOESM1_ESM.docx]

Table S1. Log-likelihood ratio tests: effect of removing fixed effect terms for linear mixed models of yield (A), PC1 (B) and PC2 (C).

| A | | | | |
| --- | --- | --- | --- | --- |
| Full model | Nested model | X^2^ | df | p |
| Variety x Environment | Variety + Environment | 2.8468 | 1 | 0.09156 |
| -332.54 | -333.96 |  |  |  |
| Variety + Environment | Environment | 6.5484 | 1 | 0.0105 |
| -333.96 | -337.24 |  |  |  |
| Variety + Environment | Variety | 20.855 | 1 | 4.953e-06 |
| -333.96 | -344.39 |  |  |  |
| B | | | | |
| Full model | Nested model | X^2^ | df | p |
| Variety x Environment | Variety + Environment | 22.271 | 1 | 2.368e-06 |
| -133.59 | -144.72 |  |  |  |
| C | | | | |
| Full model | Nested model | X^2^ | df | p |
| Variety x Environment | Variety + Environment | 2.0699 | 1 | 0.1502 |
| -118.17 | -119.21 |  |  |  |
| Variety + Environment | Environment | 90.93 | 1 | < 2.2e-16 |
| -119.21 | -164.67 |  |  |  |
| Variety + Environment | Variety | 0.8744 | 1 | 0.3497 |
| -119.21 | -119.65 |  |  |  |
