# Supplementary figures and images for: Delving deeper into technological innovations to understand differences in rice quality
Source: Rice (N Y). 2015 Jan 29;8:6. doi: 10.1186/s12284-015-0043-8 (PMC4883128; doi:10.1186/s12284-015-0043-8)

Figure S1.

A


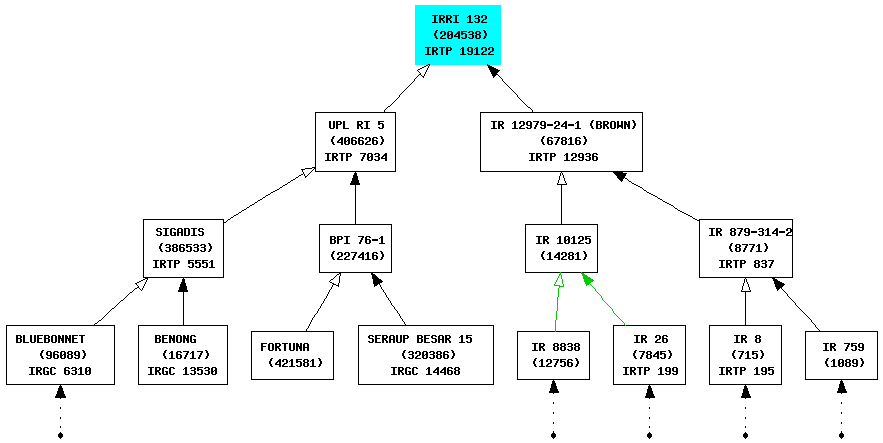


B


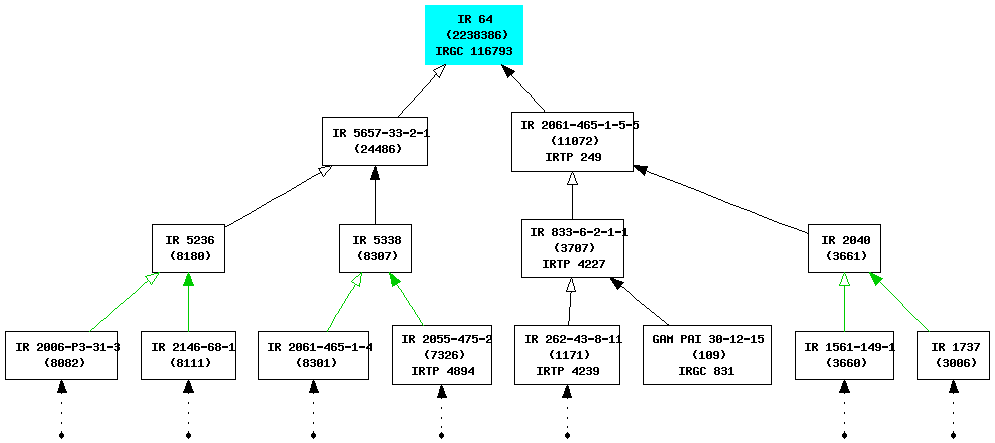

Supplement: Additional file 2: Figure S1. — Pedigree tree of (A) Apo (IRRI 132) and (B) IR64 showing no common parents in either background for three generations immediately prior to the final cross (http://www.irri.org/tools-and-databases/international-rice-information-system). [file 12284_2015_43_MOESM2_ESM.docx]
